# Takotsubo Cardiomyopathy Presenting with Sinoatrial Disease: A Rare Presentation

**DOI:** 10.7759/cureus.2743

**Published:** 2018-06-05

**Authors:** Syed Rafay Ali Sabzwari, Khurram Butt, Nimra Khan, Kailyn Mann, Tarick Sheikh, Chandra Bomma

**Affiliations:** 1 Cardiology Fellowship, Lehigh Valley Health Network, Allentown, USA; 2 Internal Medicine Residency, Florida Hospital-Orlando, Orlando, USA; 3 Medicine, Florida Hospital-Orlando, Orlando, USA; 4 Cardiology, Lehigh Valley Health Network, Allentown, USA; 5 Internal Medicine, Lehigh Valley Health Network, Allentown, USA; 6 Electrophysiology, Florida Hospital-Orlando, Orlando, USA

**Keywords:** takotsubo cardiomyopathy, sinus pause

## Abstract

Takotsubo cardiomyopathy (TCM), or apical ballooning syndrome, is a distinct nonischemic cardiomyopathy mimicking acute coronary syndrome. A 76-year-old female presented with ST elevation in the inferior lead and a troponin level of 0.81 ng/dL. An immediate coronary angiography showed non-obstructive coronary artery disease. A subsequent ventriculogram and echocardiogram showed anteroapical and distal inferior wall hypokinesis suggestive of TCM. Despite therapy with beta blocker, she was observed to have two significant sinus pauses, one eight-second, and a second 29-second pause. An urgent transvenous pacemaker was put in place and later followed by a permanent pacemaker. The patient was discharged on carvedilol and losartan. Although other arrhythmias such as complete heart block, torsades, and ventricular arrhythmias have been commonly reported, the association of TCM with recurrent sinus arrest has rarely been reported in the literature. The occurrence observed in this case implies that patients with TCM should be monitored closely for arrhythmias, and, if such a condition is identified, planning for permanent pacemaker implantation should be started early enough to avoid recurrent life-threatening episodes.

## Introduction

Takotsubo cardiomyopathy (TCM), or apical ballooning syndrome, is a distinct nonischemic cardiomyopathy mimicking acute myocardial infarction, with mid to apical left ventricular hypokinesis and basal hyperkinesis [1,2]. The incidence of TCM is highest among elderly women, and in those with history of smoking, hypertension, hyperlipidemia, and anxiety. Typically, the long-term prognosis associated with TCM is good, and often the depressed left ventricular function of TCM resolves completely over the course of several weeks to months. There have been reports of conduction abnormalities associated with this transient phenomenon including QT prolongation, torsades, ventricular tachycardia (VT), and supraventricular tachycardia [3-7]. However, atrial arrhythmias and conduction abnormalities are relatively uncommon with sinus or atrioventricular (AV) nodal dysfunction occurring in fewer than 5% of the cases [8]. This case illustrates a rare presentation of recurrent sinus arrest with TCM.

## Case presentation

**Figure 1:**
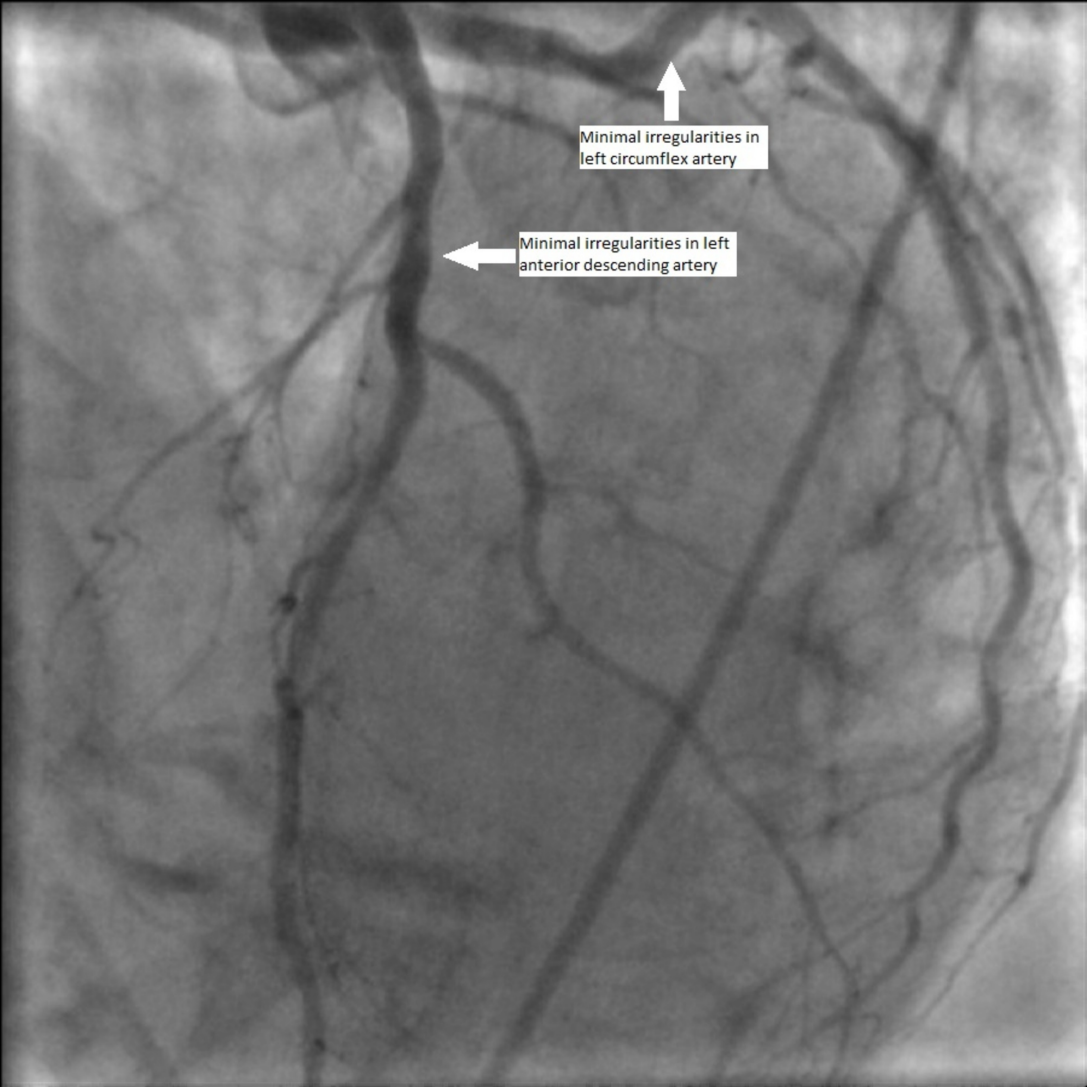
Coronary angiogram with non-obstructive coronary artery disease with 40% stenosis in the left anterior descending coronary artery.

**Figure 2:**
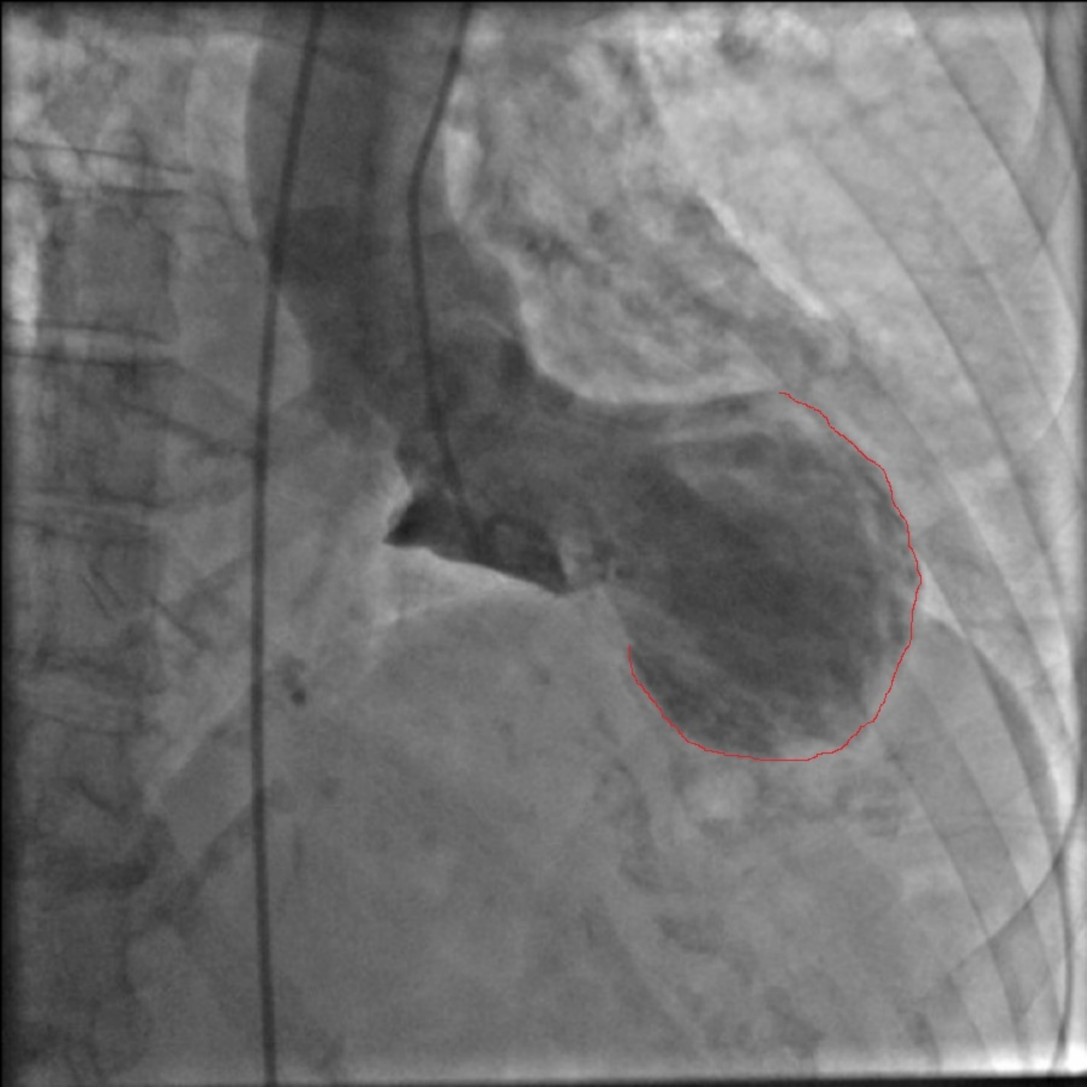
Left ventriculogram showing apical ballooning (red line).

A 76-year-old female with history of remote tobacco use, hypertension, transient ischemic attack, and osteoarthritis presented after a fall for the first time due to syncope. Her home medications included aspirin 81 mg once daily, losartan 25 mg once daily and multivitamins. On examination, there were no focal neurological deficits, no additional heart sounds or murmur were noted, and the remaining examination was unremarkable. The initial electrocardiogram (EKG) found a 2-mm ST elevation in the lateral leads I and aVL and a reciprocal 1-mm ST depression in the inferior leads II, III, and aVF. The patient’s initial troponin T level was 0.81 ng/dL (the reference range is <0.03 ng/dL). The patient immediately underwent coronary angiography due to the ST-segment elevation myocardial infarction (STEMI) alert but was found to have non-obstructive coronary artery disease (Figure 1). A left ventriculogram revealed an ejection fraction (EF) of 30% with poor anteroapical and distal inferior wall hypokinesis suggestive of Takotsubo cardiomyopathy (Figure 2).

**Figure 3:**
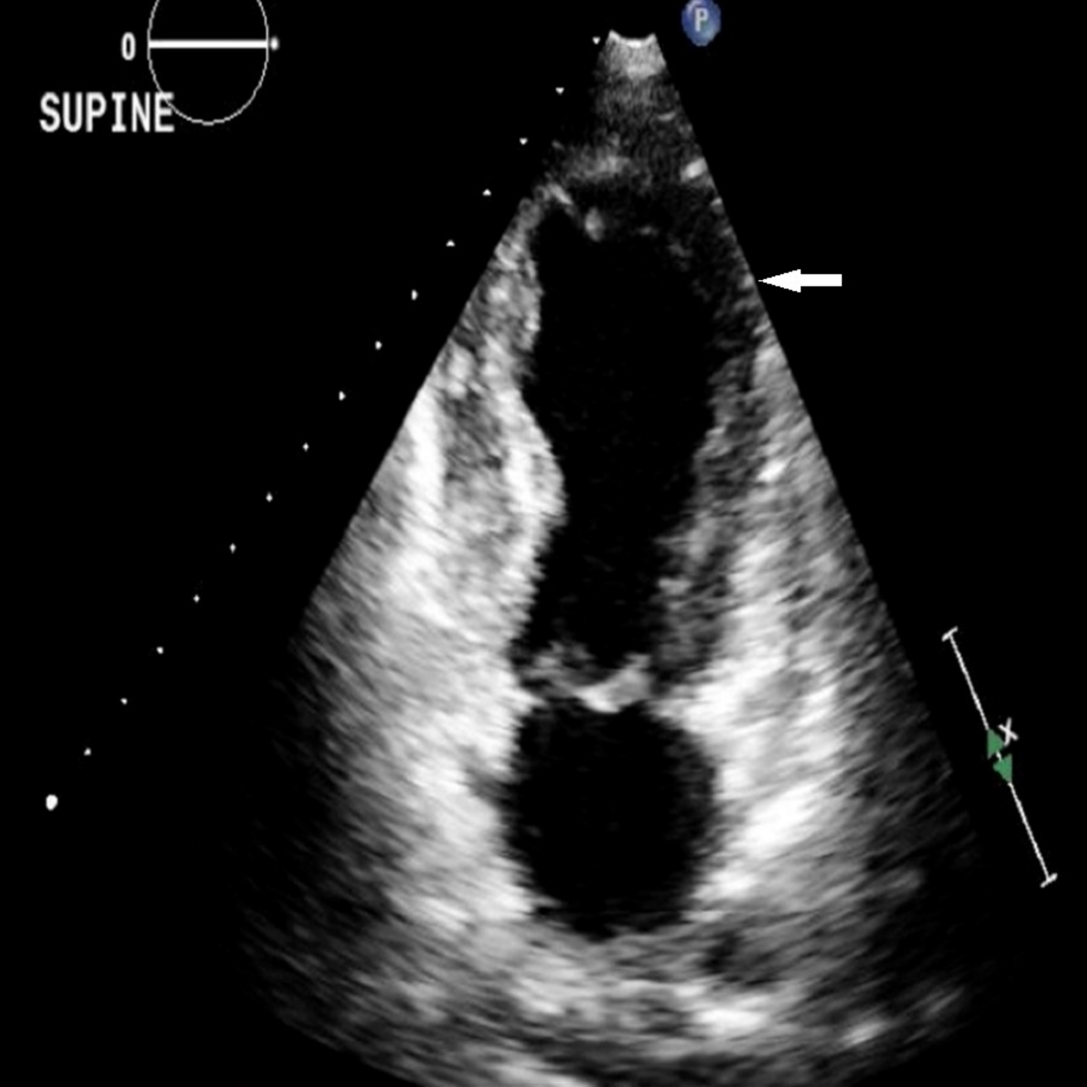
Two chamber end-systolic echocardiographic view showing apical ballooning (white arrow).

Her second set of bloodwork showed troponin levels of 0.50 ng/dL, total creatine kinase (CK) of 329 units/L (the reference range is 24 to 200 units/L) and creatine kinase-muscle/brain (CK-MB) of 27 ng/mL (the reference range is 0.1 to 6.7 ng/mL). Her lipid panel showed a total cholesterol of 140 mg/dL, low-density lipoprotein (LDL) of 60 mg/dL and high-density lipoprotein (HDL) of 73 mg/dL. A subsequent transthoracic echocardiogram confirmed the presence of apical ballooning and akinesis typical of TCM. The function of the basilar septum and the lateral basilar walls were well-preserved with an EF of less than 20% (Figure 3). In addition, she also had moderate aortic, mitral, and tricuspid regurgitation along with moderate pulmonary hypertension. A review of outside records included an echocardiogram from four months ago with an EF of 60% to 65% without any regional wall motion abnormalities, a concurrent nuclear stress test with no evidence of reversible ischemia or infarction, as well as an EKG without any pre-existing ST/T wave abnormalities.

**Figure 4:**
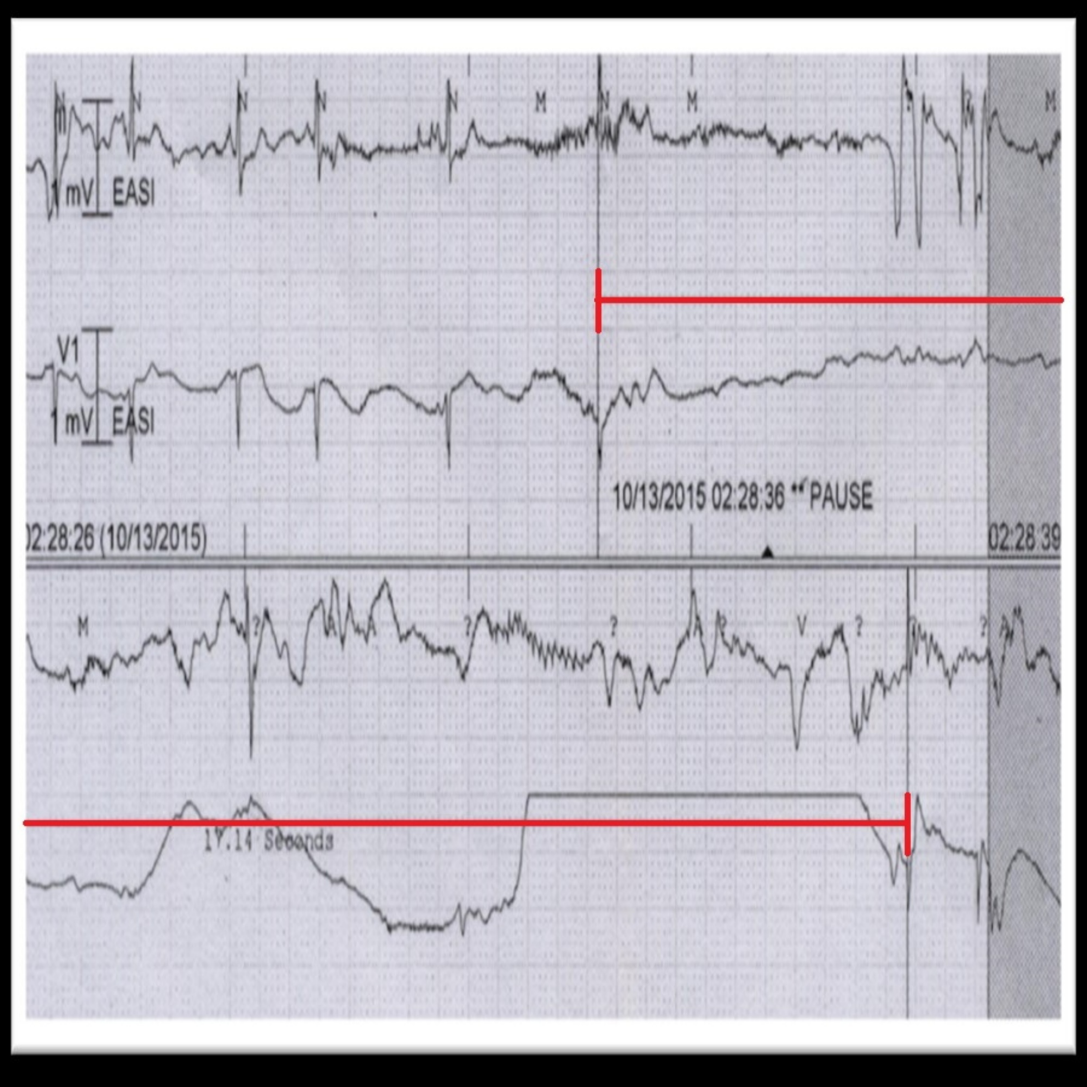
Telemetry strip showing 17 seconds sinus pause (red mark).

A day later, the patient experienced a symptomatic 17-second sinus pause that was noted on telemetry (Figure 4). The carvedilol 3.125 mg started after the angiography was performed, was discontinued. Despite this, 24 hours later the patient became unresponsive because of a 29-second sinus pause but was resuscitated by cardiopulmonary resuscitation (CPR). An emergent transvenous pacemaker was inserted due to this; it kept her hemodynamically stable after that. During the next day, the patient’s heart was paced twice by the device for eight and 14 seconds, respectively, but the patient remained asymptomatic.

As the patient required beta-blocker therapy long term to improve her cardiac function, the following day a dual-chamber permanent pacemaker was inserted, and the patient was restarted on carvedilol 3.125 mg twice daily in addition to losartan 50 mg once daily and aspirin 81 mg once daily. Two weeks after discharge on outpatient follow-up, the patient had no further recurrences of syncope. Her pacemaker was functioning normally with <1% pacing noted.

## Discussion

Takotsubo cardiomyopathy is a disease defined mainly based on the presence of ventricular wall motion disorder and is less frequently associated with conduction pathway disorders. However, TCM has also been reported to cause all degrees of AV node block, VT, and ventricular fibrillation. In the case illustrated here, a rare sinoatrial arrhythmia associated with new onset TCM was the likely etiology of her syncope. This is supported by the fact that this was the first time the patient syncopated at home while not on a beta-blocker or calcium-channel blocker. Despite that, it is widely hypothesized that TCM is secondary to catecholamine cardiotoxicity, though this does not explain the sinus arrest. There are multiple mechanisms proposed for the conduction abnormality seen in TCM. First, it is presumed that the acute atrial myopathy leads to sinus exit block. Second, episodic coronary spasms due to catecholamines can lead to episodic occurrences of such blocks as also seen in the case of vasospastic angina [9]. Third, continued ischemia leads to conduction pathway fibrosis leading to permanent conduction block [8]. In addition, age-related myopathy and damage to conduction pathways although not previously studied are also contributing factors. As this patient continued to have more frequent and longer sinus pauses, these eventually resulted in the cardiac arrest observed within one day of the diagnosis. This signifies that, in addition to patients with TCM being monitored closely for arrhythmias, if sinus arrest is identified, planning for pacemaker insertion should be begun early enough to avoid recurrent life-threatening episodes as such conduction disorders would rarely resolve in adequate time [8]. Also, conduction abnormalities can often continue to worsen in frequency, magnitude, and severity such as developing from the first-degree block to complete heart block to bundle branch blocks [4]. Recognition of this phenomenon by the cardiology community is of increasing importance. Based on reports of other arrhythmias in TCM, recovery of conduction abnormalities is usually delayed, sometimes even years after the improvement in the EF, perhaps due to abnormalities in myocardial architecture that require a long recovery time [3,4].

## Conclusions

This case highlights the importance of monitoring patients with TCM for conduction abnormalities that could be life-threatening. This is the reason why close follow-up to monitor the need for a pacemaker is important. Since there is growing evidence of various transient or permanent conduction abnormalities seen in conjunction with TCM, perhaps in the future, the diagnostic criteria for TCM will incorporate these as well.
